# Lithotomy

**Published:** 1845-01

**Authors:** 


### SELECTIONS

FROM

### AMERICAN AND FOREIGN JOURNALS.

---

*Lithotomy.*—From an interesting paper on the *Bilateral* Operation of Lithotomy, in the American Journal of the Medical Sciences, (for October,) by Professor Warren, we make the following extracts:

*Urinary Calculi rare in Boston.*—The operation of lithotomy is so rare in this place, that it would demand a longer period than the life of one practitioner to settle the question, in this part of the country, as to the best mode of operating. In the course of forty years, I have been called on to perform all the operations of lithotomy which have been done, during that period, in the city of Boston. The whole number has not exceeded twenty-five, inclusive of lithotrity cases, in a population which, during the period mentioned, has increased from about 26,000 to more than 100,000. Of the twenty-five persons thus operated on, not more than three were natives of Boston or its vicinity; the others came from the remote parts of Massachusetts, from New Hampshire, from a calcareous district in Maine, and from Nova Scotia. Perhaps it may be proper to add, that, of the cases I have operated on, two have died; one of these was a case of very unfavorable nature, the patient being of gross constitution, bad health, and having a very large stone adherent to the anterior part of the bladder; he died of suppuration in the pelvis. The other was the case of a patient who was doing perfectly well, but, on the fifth day from the operation, he indulged himself in eating heartily; he died of general peritonitis.

The bilateral operation, which Dupuytren considered more eligible than the lateral, in the estimation of Professor Warren, has the following advantages:

First, that the parts cut are more simple in structure than those concerned in the lateral operation; the nerves and the blood-vessels are necessarily smaller as they approach the median line.

Secondly, the pain and hemorrhage are, consequently, less than in the lateral operation.

Thirdly, the access to the membranous part of the urethra, and to the bladder is more direct.

Fourthly, the prostate gland being more completely exposed, may be divided with more precision.

Fifthly, the opening into the bladder may be double the size of that made in the lateral operation, without increasing the danger of cutting the vesical plexus of veins, the prostatic fascia, or the internal pudic artery.

Sixthly, the danger of subsequent inflammation is decidedly less.

The principal objection to this operation is the risk of wounding the rectum. This is certainly very considerable; but by drawing the staff with the urethra and prostate gland, towards the symphysis pubis, and with the finger of the left hand upon or in the rectum, drawing that intestine in the direction of the os sacrum, this danger will be found less, perhaps, than in the lateral operation. It may be objected, that in the present case, the wound was longer in healing than it frequently is after the lateral operation. The reason of this, however, is to be found, not in the form and direction ot the wound, or in the parts implicated, but in the morbid state of these parts, and general condition of the patient, previous to the operation.

